# Controlled clinical trial of canine therapy versus usual care to reduce patient anxiety in the emergency department

**DOI:** 10.1371/journal.pone.0209232

**Published:** 2019-01-09

**Authors:** Jeffrey A. Kline, Michelle A. Fisher, Katherine L. Pettit, Courtney T. Linville, Alan M. Beck

**Affiliations:** 1 Indiana University School of Medicine, Department of Emergency Medicine, Indianapolis, IN, United States of America; 2 Eskenazi Health, Indianapolis IN, United States of America; 3 Purdue University, Department of Comparative Pathobiology, Lafayette, IN, United States of America; VU university medical center, NETHERLANDS

## Abstract

**Objective:**

Test if therapy dogs reduce anxiety in emergency department (ED) patients.

**Methods:**

In this controlled clinical trial (NCT03471429), medically stable, adult patients were approached if the physician believed that the patient had “moderate or greater anxiety.” Patients were allocated on a 1:1 ratio to either 15 min exposure to a certified therapy dog and handler (dog), or usual care (control). Patient reported anxiety, pain and depression were assessed using a 0–10 scale (10 = worst). Primary outcome was change in anxiety from baseline (T0) to 30 min and 90 min after exposure to dog or control (T1 and T2 respectively); secondary outcomes were pain, depression and frequency of pain medication.

**Results:**

Among 93 patients willing to participate in research, 7 had aversions to dogs, leaving 86 (92%) were willing to see a dog six others met exclusion criteria, leaving 40 patients allocated to each group (dog or control). Median and mean baseline anxiety, pain and depression scores were similar between groups. With dog exposure, median anxiety decreased significantly from T0 to T1: 6 (IQR 4–9.75) to T1: 2 (0–6) compared with 6 (4–8) to 6 (2.5–8) in controls (P<0.001, for T1, Mann-Whitney U and unpaired t-test). Dog exposure was associated with significantly lower anxiety at T2 and a significant overall treatment effect on two-way repeated measures ANOVA for anxiety, pain and depression. After exposure, 1/40 in the dog group needed pain medication, versus 7/40 in controls (P = 0.056, Fisher’s exact test).

**Conclusions:**

Exposure to therapy dogs plus handlers significantly reduced anxiety in ED patients.

## Introduction

Patients in the emergency department experience both a high frequency and severity of untreated acute and chronic stress and anxiety. A convenience sample survey of 338 urban emergency department patients found that 81% of patients answered yes to the question “Have you experienced acute stress within last 3 months (a major financial, professional, or personal loss or loss/death of a loved one?).”[1] Approximately 8% of patients with low risk chest pain spontaneously reported anxiety as the primary reason for their ED visit at 90-day follow-up.[2] When surveyed prospectively in the ED, 40% of low risk chest pain patients scored >7 on the anxiety subscale of the Hospital Anxiety and Depression Survey.[3] Among 3009 ED patients with acute pain, Patel et al found the mean visual analogue scale (0–1 range) for anxiety and stress was 0.61 among women and 0.55 among men.[4] Among ED patients with asymptomatic hypertension, about 50% score in the high range (>40) on the Spielberger State Anxiety Scale.[5] Regarding the provider perception and response to patient anxiety, a national survey of 409 emergency physicians found that respondents believe that 30% of low risk chest pain had severe anxiety, but only 41% offer anxiety treatment.[6] Reasons for not treating anxiety may include the desire to avoid the side effects of anxiolytics, such as sedation, which can preclude driving home. Also, physicians are wary of generating a perception of labelling the patient as anxious, and implicitly discounting his or her reason for seeking emergency care. In this setting, therapy dogs may represent a method to address anxiety without medication, and improve the patient experience.

The rational for therapy dogs is supported by prior literature that demonstrates that human perception of stress and pain can be reduced with exposure to animals.[7–11] Other studies have found reduction in stress using therapy dogs in multiple healthcare settings.[9, 12] An average 12 minute exposure to a therapy dog reduces anxiety in 34% of fibromyalgia patients, together with reductions in pain and improvements in mood.[8] Patients with major joint replacement exposed to dog therapy required less pain medication than controls.[13] In one ED, 93% of all patients indicated desire to see a therapy dog.[14]

To our knowledge, no study has investigated the effectiveness of therapy dog in the ED setting. Accordingly, we conducted a clinical trial of dogs, compared with usual therapy, in medically stable ED patients at risk for moderate or greater anxiety. The study hypothesis was that patients exposed to a therapy dog would have lower perceived anxiety than patients with usual care while in the ED. Secondary hypotheses stated that therapy dogs would reduce perceived depression, pain and opioid requirements.

## Methods

### Overview

This was a single center, prospective controlled trial that was approved by the Indiana University School of Medicine Institutional Review Board. The trial protocol was uploaded to the clinicaltrials.gov website prior to first patient enrolled but the release of the registration was administratively delayed until after first patient was enrolled (NCT03471429). However, no portion of the protocol changed in that interval. The authors confirm that all ongoing and related trials for this drug/intervention are registered. All study procedures were performed in the emergency department at the Lois and Sydney Eskenazi hospital. The Eskenazi hospital has an existing animal therapy department, managed by a coauthor (MF). All human and animal participants were unpaid volunteers, and this study was not funded by an external source. All dogs and handlers were Therapy Certified as a team through one of the following organizations: Alliance of Therapy Dogs, Therapy dogs International, Pet Partners, Paws and Think, Love on a Leash. All dogs and handlers are registered and badge-identifiable volunteers at the hospital.

### Patient participants and trial design

Enrollment occurred during weekdays, when therapy dogs were reliably available. All patients were enrolled within two hours of arrival. The Inclusion criteria required patients to be adults, age > 18, who were awake and alert, ambulatory and without any medical emergency requiring immediate medical attention, and were not overtly intoxicated. All patients who were ultimately approached for possible participation first required that their emergency physician (either a board certified emergency physician, or a resident in training) agree with the statement: "I believe this patient is experiencing moderate or greater anxiety." At the time of this assessment, physicians were unaware of the treatment group assignment. Patients were then approached by one of two study authors (KLP or CTL) and were told of the study and its purpose and screened for exclusion criteria: agitation that precluded normal conversation, fear of dogs, dog bite, allergy to dogs. We recorded the number of patients who met one of these criteria and were willing to participate. We sought to study patients assigned to either a dog or usual care under the most similar clinical conditions as reasonably possible. Accordingly, we studied two patients temporally as close as possible in the same unit in the emergency department, one patient randomly assigned to a dog plus handler and the other to usual care. The choice as to which came first was by a preprinted random schedule (StatsDirect Statistical Software, v 3.0.187, Cheshire, England). The large patient volume at Eskenazi hospital, coupled with the high frequency of patients who qualified, allowed us to enroll one patient in each group within ~1 hour of each other on the same shift, such that both patients were studied in the same location, with the same nursing, ancillary and physician staff. Patients allocated to the control group were told at the start of the consent process that they would not see a dog. Patients allocated to the therapy dog group were then exposed to a dog plus a handler. The handlers then escorted the dog into the patient room. Handlers were trained to use a script to introduce themselves and the dog, but were free to “ad-lib” the conversation. Dogs remained on a 5 feet long leash held by the handler during the entire encounter. Patients were freely able to touch or pet the dog if they wished. All patient rooms contained only one patient. Family or visitors were allowed to remain in the room. The dog and handler remained in the room for 15 minutes. To reduce interruptions, research personnel placed a sign on the door stating “Therapy dog session in progress for 15 minutes”. Control subjects were allowed to remain in their rooms as usual care processes proceeded. Research personnel, working together with dog handlers, took precautions from preventing the control subjects from accidentally seeing a therapy dog. On T2 measurement, controls were asked if they saw a dog. Data were then collected from four sources: 1. The patient, 2. The medical record, 3. The primary physician caring for the patient, and 4. The dog handlers.

Patients provided the data needed for the primary and secondary outcomes using anxiety, depression and physical pain assessments on a 0–10 point FACES scale using three instruments shown in the appendix. This scale is simple to understand, and visually based, requiring no literacy. This scale has been validated in other populations and appears to have similar construct validity to more complex tools.[15–17]

The primary outcome measure was the patient-reported anxiety score (0–10: 0 = balanced mood [least anxiety], 2 = slight fear and worry, 4 = mild fear and worry, 6 = moderate worry, physical agitation, 8 = feeling really bad, at the edge, 10 = out of control behavior, self-harm [worst anxiety]) Secondary outcomes included change in pain score (0–10: 0 = no hurt [least pain], 2 = hurts little bit, 4 = hurts little more, 6 = hurts even more, 8 = hurts whole lot, 10 = out of control pain with feelings of self-harm [hurts worst]) and change in depression score (0–10): 0 = balanced mood [least depressed], 2 = slightly depressed, 4 = mildly depressed, 6 = definite malaise, 8 = feeling really bad, at the edge, 10 = despair, suicidal feelings [worst depressed]).

These three measurements (anxiety, pain,and depression) were obtained three times: at baseline (T0, prior to dog or control), and then again, about 30 min after after exposure to the dog or usual care (T1), and then as late as possible prior to patient discharge (T2). Patients with a 0 score on anxiety were screen failures. The medical record was reviewed and data were abstracted by study personnel to determine patient demographics, vital signs, past medical and psychiatric history, and all medications and their times of order in the ED. All clinical data were recorded in the ED on the same day of enrollment, and were supplemented by queries to the patients and providers as needed. Physicians, blinded to the patients’ self-assessments, were asked to rate their patients’ anxiety, depression and pain on the same scales as were used by the patient prior to intervention. The dog handlers were asked to provide written field notes with instructions to record their impressions of the patient’s words, affect and behaviors before during and after interaction with the dog. Handlers were encouraged to report without bias, both negative or positive observations, and to report both direct observations of behavior, and also their own interpretations of the patient’s mood, affect or emotional state.

### Sample size computation

The primary outcome was patient reported anxiety at T1, with the assumption that data would be normally distributed and an unpaired t-test would be applicable. Extrapolating from prior work by Barker et al and Marcus, et al we set the clinically significant reduction in anxiety as requiring a greater than 2 point (20%) decrease in anxiety at T1 compared with usual care, expecting a standard deviation of 3.[8, 9] With α = 0.05 and β = 0.20, this required 37 pairs. Accordingly, the sample was set at 40 per group with complete data.

### Data analysis

All data from patients, providers and the medical record were recorded in the REDCap data archiving system.[18] Data from the scales were analyzed for normality and all anxiety data were found to pass the D'Agostino & Pearson test (P>0.05). Accordingly, we report P values for paired t-testing (T0 vs. T1) and and unpaired t-testing (T1 vs T1 and T2 vs. T2) for each group. We also compare medians at individual times are compared between patients receiving a therapy dog and usual care using the Mann-Whitney U test. The overall effect of treatment on repeated measures (T1 and T2) were assessed using a two-way repeated measures analysis of variance with the group P value as the primary test of importance. Prior to applying the we tested for symmetry (kurtosis and skewness between -2 and +2), equal variances(F test>0.05). Greenhouse Geisser correction for sphericity. Another preplanned objective was to measure the frequency of opiate drug prescriptions in the ED after exposure. These frequencies were compared between groups using Chi Square and Fisher’s exact test. All data were plotted using GraphPad Prism version 7.00 for Windows (GraphPad Software, La Jolla California USA). Statistical analysis were performed using StatsDirect (v.3.0.187, Cheshire, England).

Field notes from dog handlers were independently read by two team members, and analyzed by In-commercial software (Nvivo Version 12.0.0.71, QSR International) to parse into phrases and sentences that were theme-coded using a focused coding approach with special attention to predefined meanings: changes in patient verbal communication, changes in patient affect and changes in patient behavior from the start of the session to the end of the session.[19] We also recorded the identity and photographs of the dogs to allow secondary computation of individual dog performance.

## Results

**Fig 1:**
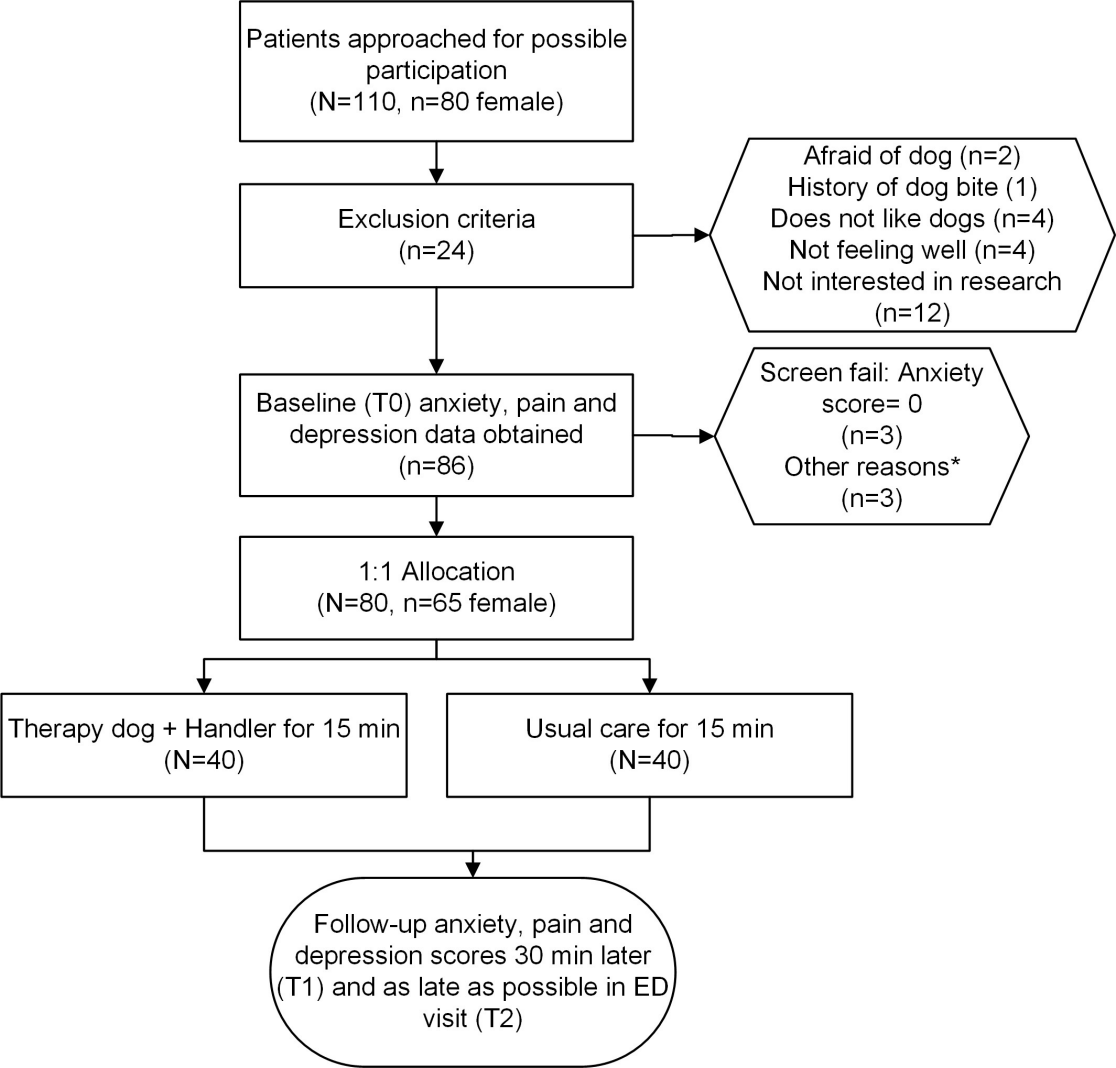
Consort flow diagram. Flow diagram of patients approached for participation *Reasons for voluntary withdrawal: 1. Patient was worried that police would come in with the dog; 2. Likes dogs, but not for her. Thinks therapy dogs would help people though; 3.Goes to therapy every other week and there is a dog there, but didn't want to see one today.

Fig 1 presents the Consort flow diagram for the study. We approached 110 patients, and 17 stated immediately they were not interested in research or not feeling well enough to participate. Among the remaining 93, 7 (8%) had direct dog-related exclusions because of fear of dogs (n = 2), history of dog bite (n = 1), or dislike of dogs (n = 4), leaving 86 patients, of whom 3 more were excluded because they reported an anxiety score of 0 and three more declined for reasons detailed in the figure legend. Table 1 compares the demographic data, and chief complaints. The salient finding was that 81% of patients who were enrolled were female. Among the 24 patients who were screened but not enrolled, 15/24, 63% were female.

**Table 1:** Demographic features and chief complaints of patient participants.

Demographic data	Therapy dog and handler (n)	% of 40	Usual care (n)	% of 40	P Value**
Age >60	7	18%	7	18%	0.259
Age 40–60	16	40%	12	30%	0.999
Age <40	18	45%	22	55%	0.275
Mean age (standard deviation, sd)	46	sd 16	42	sd 15	0.253†
Female gender	34	85%	31	78%	0.41
White race	25	63%	20	50%	0.271
Employed full or part time	14	35%	18	45%	0.374
No High School diploma	15	38%	11	28%	0.353
High School diploma or GED	15	38%	17	43%	0.657
Some College	7	18%	9	23%	0.591
College diploma	3	8%	3	8%	0.999
Married	6	15%	9	23%	0.41
Disabled	3	8%	2	5%	0.999
Chief complaint					
Abdominal pain	2	5%	3	8%	0.999
Anxiety	3	8%	3	8%	0.999
Chest pain	8	20%	7	18%	0.999
Other painful condition	6	15%	6	15%	0.999
Psychiatric clearance or evaluation*	9	23%	9	23%	0.999
Shortness of breath	4	10%	3	8%	0.999
Other complaints	8	20%	9	23%	0.999

*Includes Drug overdose (n = 1 in each group) and suicidal ideation (n = 2 in each group)

**Exact binomial for independent proportions

†Unpaired t-test

Table 2 compares past medical and psychiatric diagnoses between the patients assigned to a therapy dog or usual care. More patients assigned to the therapy dog group had diabetes. Ninety percent of our patients enrolled had no family or friends present during their entire ED stay. One salient finding was the unexpected preponderance of female participants in the entire study.

**Table 2:** Past medical and psychiatric diagnoses.

Medical diagnoses	Therapy dog and handler (n)	% of 40	Usual care (n)	% of 40	P Value*
No medical diagnosis	3	8%	4	10%	0.999
Asthma	8	20%	7	18%	0.999
Chronic obstructive pulmonary disease	9	23%	3	8%	0.07
Current cigarette use	19	48%	21	53%	0.999
Coronary artery disease	6	15%	0	0%	0.013
Diabetes Mellitus	11	28%	2	5%	0.007
Hypertension	17	43%	15	38%	0.99
Stroke	1	3%	1	3%	0.999
Kidney disease	2	5%	0	0%	0.248
HIV	1	3%	0	0%	0.999
Psychiatric diagnoses					
No psychiatric diagnosis	22	55%	18	45%	0.383
Attention deficit disorder	1	3%	1	3%	0.999
Anxiety	9	23%	13	33%	0.332
Bipolar	3	8%	6	15%	0.318
Depression	11	28%	13	33%	0.636
Schizophrenia	1	3%	1	3%	0.999
Post traumatic stress disorder	1	3%	2	5%	0.999

*Exact binomial for independent proportions

### Physician assessments compared with patient self-assessments

All patients were thought to have “moderate or greater” anxiety by their care providers, whose gender and training level of care providers are presented in Table 3. In only 3 out of 84 cases (4%) did the patient mark a 0 on the anxiety scale, leading to screen failure. Comparison of physician baseline estimates of anxiety, pain and depression with patient self-estimates revealed no significant difference for anxiety (P = 0.77, paired t-test), or depression (P = 0.25, paired t-test). However, physicians had significantly lower estimates of pain (mean anxiety estimate for physicians = 4.3, sd 3.1, versus mean anxiety estimate for patients = 6.1, sd 3.3; P = 7x10^-9^, paired t-test).

**Table 3:** Provider characteristics.

	dog	%	no dog	%	P Value*
Male	17	43%	15	38%	0.657
Attending	10	25%	13	33%	0.473
Resident	23	58%	19	48%	0.383
Advanced practitioner	11	0.28	9	23%	0.618

*Exact binomial for independent proportions

### Effect of therapy dogs + handlers

**Fig 2:**
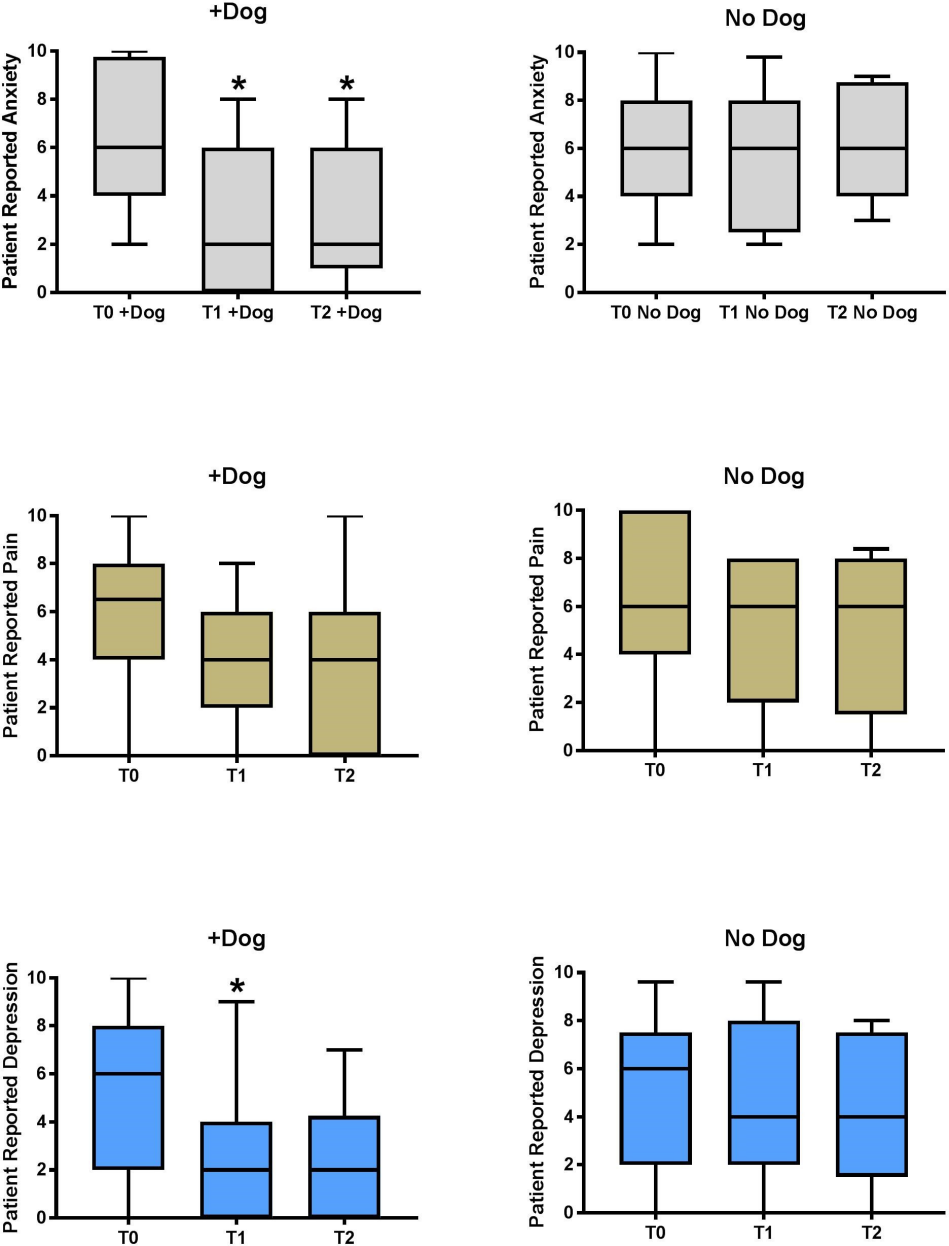
Main findings. Comparison of median and interquartile ranges (top and bottom of boxes) and 90^th^ percential ranges (whiskers) of patient reported anxiety, pain and depression reported by patients assigned in a 1:1 ratio to a therapy dog plus handler (+Dog) or usual care (No Dog). T0 was measured at baseline prior to exposure; T1 was at 30 min after exposure, and T2 was made approximately one hour later. *P<0.05 by Unpaired t-test and Mann-Whitney U at the time point). All three measurements (anxiety, pain and depression) were significantly different for treatment effect (Dog vs. No Dog) by repeated measures analysis of variance).

Fig 2 presents the main finding of the study. Compared with usual care, exposure to a therapy dog plus handler was associated with a 35% reduction in patient reported anxiety state at T1 (30 min after departure of dog and handler), and this reduction was sustained until T2. The median time differences for T2-T1 measurement for the dog + handler group was 78 minutes (1^st^-3^rd^ quartile range 68–105 minutes) compared with 91 minutes (1^st^-3^rd^ quartile range 76–111 minutes) in the usual care group. Fig 2 also shows that with usual care, patient reported levels of anxiety and depression remained constant throughout their ED stay, whereas after exposure to the dog + handler, both of these measurements decreased. The overall treatment effect of dog + handler on anxiety was also significantly different repeated measures ANOVA, (F(1,80) = 13.7; treatment effect P = 0.0003 and time*group interaction P = 0.03). The relative therapeutic effect of dog+handler was not as prominent for patient reported pain and depression, although T1 was significantly lower for depression (P<0.05 by Mann-Whitney U and unpaired t-test). Moreover, repeated measures ANOVA demonstrated that the overall treatment effect was significant for dog + handler compared with usual care by for both pain [F(1, 80) = 12.4, P = 0.0008], and depression [F(1,80) = 6.1, P = 0.014]; the P value from the time*group interaction was not significant (P>0.05) for either pain or depression. S1, S2 and S3 Tables (anxiety, pain and depression, respectively), show P values for paired t-test comparing T0 ato T1 and T2, and P values from both the Mann-Whitney U test and unpaired t-test between groups for T0, T1 and T2.

No patient in either group received any medication between T0 and T1. However, between T1 and T2, 1/40 patients exposed to a therapy dog and handler were prescribed an opioid pain medication while in the emergency department, compared with 7/40 with usual care (P = 0.02, Chi Square; P = 0.057, Fisher’s Exact test). Regarding anxiolytic agents, in the dog group, one patient received lorazepam and one received chlordiazepoxide, both by mouth after enrollment. In the control group, four patients received lorazepam, two received hydroxyzine and one received haloperidol, all parenterally administered.

We examined changes in anxiety based upon chief complaint groupings that allowed at least n = 6 measurements at T1 and T2. The mean decrease in anxiety for patients exposed to dogs + handlers was 45±28%, 48±24% and 63±30% for complaints of chest pain, other pain and psychiatric complaints, respectively. The corresponding changes in anxiety for patients with usual care were 10±5%, 4±2%, 8±3% for complaints of chest pain, other pain and psychiatric complaints, respectively.

Seventeen therapy dogs and 12 handlers participated. To examine for potential variation based upon dog-handler combinations, we calculated the median of the differences between T2-T0 for the anxiety data for each dog-handler combination (S4 Table). There were only two instances in which a unique dog and unique handler had more than two patient encounters to allow statistical comparison (P = 0.64, by Mann Whitney U).

Field notes were provided by nine handlers. Focused thematic analysis by Nvivo revealed four dominant themes characterizing the behavior changes of interest with the number of instances in parenthesis representing unique patients: 1. change in verbal expression (5), 2. change in behavior (2), 3. change in affect (8), and 4. change in mood (8) All changes were considered to represent positive emotional changes in the opinon of the handlers. Table 4 presents key excerpts provided by the handlers describing each of these behavior changes they observed with the therapy dog visit.

**Table 4:** Excerpts from field notes of therapy dog handlers regarding patient behaviors.

Behavior	Excerpt
Change in verbal expression	“He slowly began to make more eye contact with me and engage in our conversation…”
	“The patient reached out to pet Cali and almost immediately changed from crying out loud to presenting a calm, inquisitive voice asking about Cali.”
Change in behavior	“The patient ceased shouting and rolling head from side to side”
	“Within minutes, the patient went from being balled up on the stretcher, rocking back and forth, to on his hands and knees on the floor, playing with the dog.”
Change in Affect	"The patient’s demeanor had changed from being physically and emotionally stressed to laughing and enjoying both Cali’s and my presence”
	"She had tears on her cheeks, and since she missed her own dog terribly, for that brief time a hole was filled.”
Change in mood	“The whole mood changed and everyone relaxed.”
	“Overall I saw positive interest in the dogs and a calming effect of the dog visits.”
	"With family’s approval, patient and dog were happily relaxing together and it was evident to all those present (including the nurses) that this was the best thing that could have happened for that patient. "

## Discussion

This clinical trial demonstrates novel evidence that animal assisted therapy can reduce patient perception of anxiety in the emergency care setting. We found that compared with usual care, when anxious patients were subjected to a 15-min exposure to a certified therapy dog and handler, they reported a statistically significant and sustained 35% decrease in anxiety and an overall significant decrease in patient reported pain and depression. No patient terminated the session early, and only one of 40 patients in the therapy dog group had an increase in anxiety, compared with four of 40 in the usual care group. Moreover, only 2.5% of patients exposed to the dogs receive opioid pain medication during the remainder of their ED stay compared with 17.5% of usual care patients (P = 0.056 by Fisher’s exact test). Examination of the handlers’ field notes reveal additional qualitative insights into the powerful effect of the human-animal interaction on anxious patients in the ED setting. The overarching theme was that dogs consistently elicited a change in body posture, and converted patient affect from negative to positive, and cause many patients to physically open their posture, pet the dog and even play with the dog.

We believe this to be the first controlled clinical trial of animal assisted therapy in the ED setting to measure patient reported outcomes. Prior literature has demonstrated a positive role of therapy dogs on anxiety and pain in dental clinics, psychiatric wards, clinics, rehabilitation units, and pediatric wards.[7–9, 12, 20, 21] Only one study has reported data regarding therapy dogs in the ED setting; that study used survey methodology and found a high level acceptability by patients and providers, but the report did not provide patient reported or care process measurements.[14] Although the mechanism by which dogs reduce human anxiety remains uncertain, the biophilia hypothesis provides one explanation. The biophilia hypothesis states that that human gaze and attention on animals elicits a calming effect on the human autonomic nervous system, manifested as lower heart rate, blood pressure and vasodilation.[20–23] The hypothesis further states that humans have “the urge to affiliate with other forms of life,” a statement that resonates in the context of the ED, where anxious patients are often left alone in rooms for hours.[24]

The ED simultenously represents an environment that has great need for the potential benefits of animal assisted therapy, but also presents unique challenges to its implementation. The availability of certified therapy dogs remains speculative; one advocacy group estimates the number of registered therapy dog-handlers at about 25,000.[25] Most handlers (and dogs) are volunteers who are willing to work for nothing (except an occasional treat). Similar to prior work, we found that 92% of ED patients were interested in seeing a therapy dog.[14] By comparison, slightly fewer, 98/110 (88%) were willing to participate in research. At the same time, many emergency care patients yearn for affective reassurance, and unconditional attention and concern for them as a whole person.[26, 27] At least 40% of ED patients with medically unexplained or chronic pain have moderate to severe situational anxiety, and a larger fraction admit to severe chronic stress.[1, 3, 28] Challenges to initiating therapy dogs include the administrative hurdles required to obtain hospital credentials for therapy dogs and handlers to visit the ED, including “worst-case” concerns over possible patient phobias for dogs, allergies, zoonotic infections, and hygiene. Other challenges include the fact that dogs and handlers are not uniformly available, yet EDs are always open. Although data are lacking, we speculate that therapy dogs are most prevalent in metropolitan areas, and during daytime hours or evenings, whereas many patients with acute stress present at night as well as in rural hospitals.

### Limitations

First, this work is primarily hypothesis-generating inasmuch as this design does not provide a psychological or biological mechanism to explain why patients had lower anxiety, pain and depression.[29] The beneficial effect could have been a result of distraction from negative feelings by the emotional affection and physical touch with the dog, or from human companionship, given that the handlers had some degree of conversation with all patients. Second, although it was not possible to document the degree of conversation held by patients with usual care, we believe it is highly probable that most of them had limited to no interaction with other humans during the 15 minutes without a dog. We did not ask handlers or research associates to provide a checklist or scale to assess their opinions of the degree of interaction shown by the patient with the dog or the handler. Another unmeasured variable is the degree to which patients in the control group may have experienced disappointment at being in a group that did not see a dog, which could have worsened their mood. The finding that 81% of participants were females was unexpected and may imply that the benefit of therapy dogs to treat anxiety is somewhat gender specific. We are uncertain whether this female predominance reflects clinical truth (meaning more female patients were anxious than male patients), or if it were an artifact of emergency physicians’ tendency to over-recognize female patients as anxious. Forty-nine percent of all patients in the Eskenazi ED were males during the time of the study. Of relevance, female patients generally tend to score higher on state anxiety scales,[17] and a prior study found that 72% of ED patients with low-risk chest pain and high anxiety were women.[3]

## Conclusion

The majority of ED patients with suspected anxiety were interested in seeing a therapy dog. Exposure to a therapy dog and handler significantly lowered anxiety, pain and depression scores in ED patients whom physicians thought had moderate or severe anxiety. These data support the use of therapy dogs to alleviate anxiety in ED patients with suspected anxiety.

## Supporting information

S1 File Study information sheet. (PDF)

S2 File IRB letter. (PDF)

S3 File Data collection form. (PDF)

S4 File Consort. (DOC)

S5 File Study protocol. (PDF)

S1 Table P values for anxiety scores. (DOCX)

S2 Table P values for depression scores. (DOCX)

S3 Table P values for pain scores. (DOCX)

S4 Table Performance of each therapy dog and handler. (DOCX)
