# Supplementary material for: Controlled clinical trial of canine therapy versus usual care to reduce patient anxiety in the emergency department
Source: PLoS One. 2019 Jan 9;14(1):e0209232. doi: 10.1371/journal.pone.0209232 (PMC6326463; doi:10.1371/journal.pone.0209232)
Supplement: S1 File — (PDF) [file pone.0209232.s001.pdf]

INDIANA UNIVERSITY STUDY INFORMATION SHEET FOR

Canine-Assisted ANxiety reduction IN Emergency care (CANINE)

You are invited to participate in a research study of dog-assisted anxiety reduction in the emergency department. You were selected as a possible subject because there is a possibility you are feeling anxious. We ask that you read this form and ask any questions you may have before agreeing to be in the study.

The study is being conducted by Dr. Jeffrey Kline, MD, Indiana University School of Medicine Emergency Medicine.

STUDY PURPOSE

The purpose of this study is to see if dogs can reduce the anxiety level of patients in the emergency department.

PROCEDURES FOR THE STUDY:

If you agree to be in the study, you will do the following things:

Score your pain, anxiety, and depression on the provided forms. A dog will then come in to your room for approximately 15 minutes after which you will score your pain, anxiety, and depression again.

RISKS AND BENEFITS

There is a small risk that you may feel uncomfortable scoring your pain, anxiety, and depression. If at any point, you wish to not answer a question, you may do so.

The possible benefits of participating in this research are the potential of anxiety reduction after your visit with the dog.

CONFIDENTIALITY

No personally identifying information will be collected so there will be no risk of loss of confidentiality related to this study.

PAYMENT

You will not receive payment for taking part in this study.

CONTACTS FOR QUESTIONS OR PROBLEMS

For questions about the study, contact the researcher Dr. Jeffery Kline at 317-880-3869.

For questions about your rights as a research participant or to discuss problems, complaints or concerns about a research study, or to obtain information, or offer input, contact the IU Human Subjects Office at (317) 278-3458.

VOLUNTARY NATURE OF STUDY

Taking part in this study is voluntary. You may choose not to take part or may leave the study at any time. Leaving the study will not result in any penalty or loss of benefits to which you are entitled. Your decision whether or not to participate in this study will not affect your current or future relations with Eskenazi Health.
