# Supplementary material for: Controlled clinical trial of canine therapy versus usual care to reduce patient anxiety in the emergency department
Source: PLoS One. 2019 Jan 9;14(1):e0209232. doi: 10.1371/journal.pone.0209232 (PMC6326463; doi:10.1371/journal.pone.0209232)
Supplement: S2 File — (PDF) [file pone.0209232.s002.pdf]

To: Jeffrey Kline
EMERGENCY MEDICINE

From:

Chair - IRB-01
Human Subjects Office
Office of Research Compliance – Indiana University

Date: December 21, 2017

RE: **NOTICE OF EXEMPTION - NEW PROTOCOL**

Protocol Title: Canine-Assisted ANxiety reduction IN Emergency care (CANINE)

Study #: 1712453251

Funding Agency/Sponsor: None

Status: Exemption Granted | Exempt

Study Approval Date: December 21, 2017

The Indiana University Institutional Review Board (IRB) IRB00000220 | IRB-01 recently reviewed the above-referenced protocol. In compliance with (as applicable) 45 CFR 46.109 (d) and IU Standard Operating Procedures (SOPs) for Research Involving Human Subjects, this letter serves as written notification of the IRB's determination.

Under 45 CFR 46.101(b) and the SOPs, as applicable, the study is accepted as Exempt (700) Category 6 Flex: Research that is not federally funded or otherwise federally regulated and involves collection of data from voice, video, digital, or image recordings made for research purposes. (710) Category 7 Flex: Research that is not federally funded or otherwise federally regulated and is on individual or group characteristics or behavior (including, but not limited to, research on perception, cognition, motivation, identity, language, communication, cultural beliefs or practices, and social behavior) or employs survey, interview, oral history, focus group, program evaluation, human factors evaluation, or quality assurance methodologies., with the following determinations:

- **Waiver of documentation of informed consent under 45 CFR46.117(c)**

Acceptance of this study is based on your agreement to abide by the policies and procedures of the Indiana University Human Research Protection Program and does not replace any other approvals that may be required. Relevant policies and procedures governing Human Subjects Research can be found at: http://researchcompliance.iu.edu/hso/hs_guidance.html.

The Exempt determination is valid indefinitely. Substantive changes to approved exempt research must be requested and approved prior to their initiation. Investigators may request proposed changes by submitting an amendment through the KC IRB system. The changes are reviewed to ensure that they do not affect the exempt status of the research. Please check with the Human Subjects Office to determine if any additional review may be needed.

You should retain a copy of this letter and all associated approved study documents for your records. Please refer to the assigned study number and exact study title in future correspondence with our office. Additional information is available on our website at at http://researchcompliance.iu.edu/hso/.

If your source of funding changes, you must submit an amendment to update your study documents immediately.

If you have any questions or require further information, please contact the Human Subjects Office via email at irb@iu.edu or by phone at 317-274-8289 (Indianapolis) or 812-856-4242 (Bloomington).
