# Supplementary material for: Controlled clinical trial of canine therapy versus usual care to reduce patient anxiety in the emergency department
Source: PLoS One. 2019 Jan 9;14(1):e0209232. doi: 10.1371/journal.pone.0209232 (PMC6326463; doi:10.1371/journal.pone.0209232)
Supplement: S3 File — (PDF) [file pone.0209232.s003.pdf]

Demographics and History

Record ID	______________________
Age	______________________
Gender	◯ Female<br>◯ Male
Race	◯ American Indian or Alaskan Native<br>◯ Asian<br>◯ Native Hawaiian or other Pacific Islander<br>◯ Black or African-American<br>◯ White<br>◯ More than one race<br>◯ Other
Race - Other	______________________
Hispanic or Latino origin?	◯ Yes<br>◯ No
Employment status	◯ Employed at a job, full-time<br>◯ Employed at a job, part-time<br>◯ Homemaker, not currently working for pay<br>◯ Not currently employed, retired<br>◯ Not currently employed, not retired
Marital Status	◯ Single<br>◯ Married<br>◯ Separated<br>◯ Divorced<br>◯ Widowed
What is the highest level of education that you completed?	◯ Some High School<br>◯ High School<br>◯ GED<br>◯ Some College<br>◯ Graduated College<br>◯ Graduate/Professional School
Medical History	☐ None<br>☐ Asthma<br>☐ COPD<br>☐ Smoker<br>☐ Coronary Artery Disease (CAD)<br>☐ Diabetes Mellitus<br>☐ Hypertension<br>☐ Prior stroke<br>☐ Chronic kidney disease<br>☐ Hyperlipidemia<br>☐ HIV<br>☐ Other
Medical History - Other	______________________

Psychiatric History

- [ ] ADHD
- [ ] Anxiety
- [ ] Bipolar Disorder
- [ ] Borderline Personality Disorder
- [ ] Depression
- [ ] Schizophrenia
- [ ] Other

Psychiatric History - Other ______________________________

ED Data

Group ◯ Dog ◯ No Dog

Time of verbal consent ________________________

Chief Complaint ________________________

Provider Name ________________________

Provider Status ◯ Attending ◯ Resident ◯ Advanced Practitioner

---

Vital Signs

Temperature (C) ________________________

Heart Rate ________________________

Respiratory Rate ________________________

SpO2 ________________________

Systolic BP ________________________

Diastolic BP ________________________

Medication

Medication Name ________________________

Time ________________________

Dose ________________________

Route of administration ________________________

Additional med? ◯ Yes ◯ No

Medication Name ________________________

Time ________________________

Dose ________________________

Route of administration ________________________

Additional med? ◯ Yes ◯ No

Medication Name ________________________

Time ________________________

Dose ________________________

Route of administration	__________________
Additional med?	◯ Yes<br>◯ No
Medication Name	__________________
Time	__________________
Dose	__________________
Route of administration	__________________
Additional med?	◯ Yes<br>◯ No
Medication Name	__________________
Time	__________________
Dose	__________________
Route of administration	__________________

Pain/Depression/Anxiety Data

Timepoint ◯ Baseline (T0)
◯ T1

Time Scales Administered ______________________________

Time dog exits room ______________________________

Pain Scale

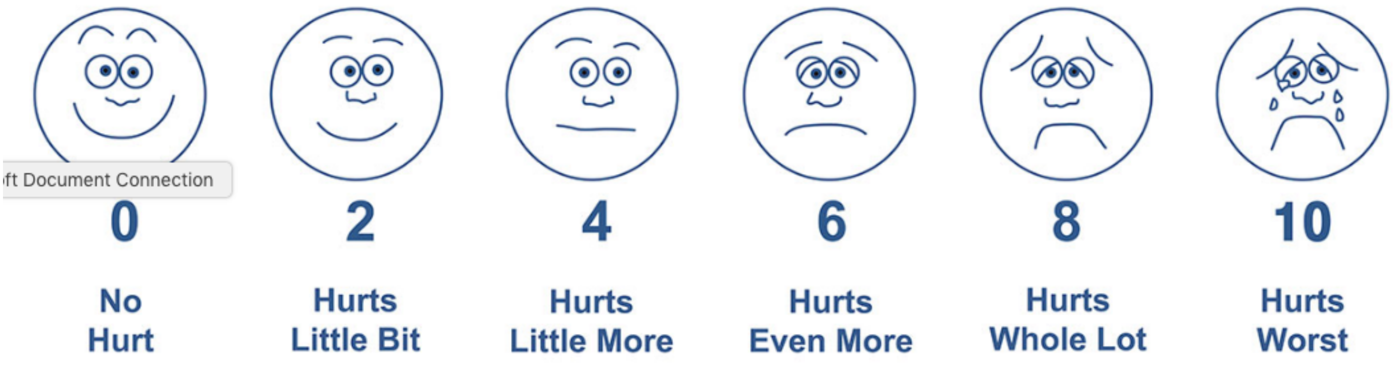

Provider
Pain rating based on image above

0 10

(Place a mark on the scale above)

Patient
Pain rating based on image above

0 10

(Place a mark on the scale above)

Depression Rating Scale

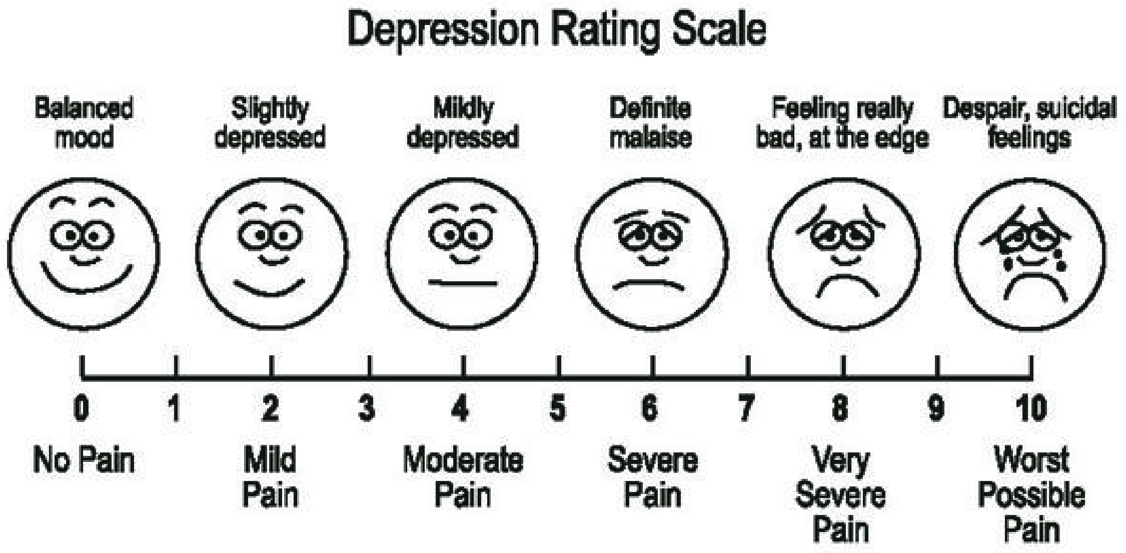

Provider
Depression rating based on image above

0 10

(Place a mark on the scale above)

Patient
Depression rating based on image above

0 ________ 10

(Place a mark on the scale above)

Anxiety Rating Scale

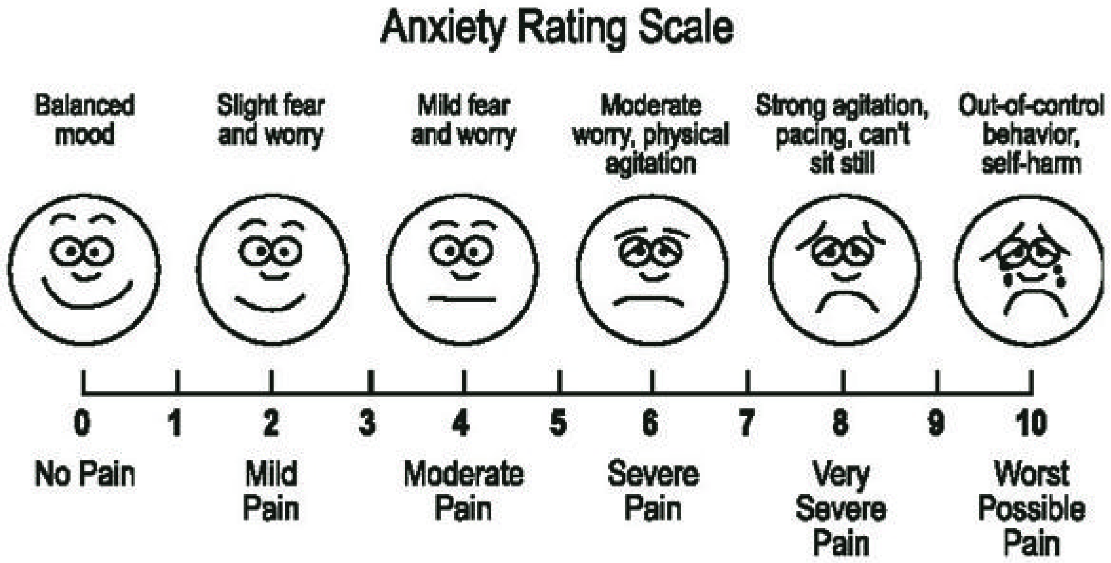

Provider
Anxiety rating based on image above

0 ________ 10

(Place a mark on the scale above)

Patient
Anxiety rating based on image above

0 ________ 10

(Place a mark on the scale above)

Time dog enters room ____________________
