# Supplementary material for: Controlled clinical trial of canine therapy versus usual care to reduce patient anxiety in the emergency department
Source: PLoS One. 2019 Jan 9;14(1):e0209232. doi: 10.1371/journal.pone.0209232 (PMC6326463; doi:10.1371/journal.pone.0209232)
Supplement: S5 File — (PDF) [file pone.0209232.s005.pdf]

Canine-Assisted ANxiety reduction IN Emergency care (CANINE)

Jeffrey A. Kline MD

Principal investigator

12-17-2017

Table of Contents:

Study Schema

1.0 **Background**
2.0 **Rationale and Specific Aims**
3.0 **Inclusion/Exclusion Criteria**
4.0 **Enrollment/Randomization**
5.0 **Study Procedures**
6.0 **Reporting of Adverse Events or Unanticipated Problems involving Risk to Participants or Others**
7.0 **Study Withdrawal/Discontinuation**
8.0 **Statistical Considerations**
9.0 **Privacy/Confidentiality Issues**
10.0 **Follow-up and Record Retention**

SIGNIFICANCE

Prior literature demonstrates that human stress can be reduced with exposure to animals.(1-3) Other studies have found reduction in stress using therapy dogs in multiple healthcare settings.(3, 4) Interaction with therapy dogs may reduce anxiety and psychological distress, leading to lower need for pain medicaition. (2, 5)

RATIONALE

This study challenges current dogma by introducing a widely available, low cost method of dog therapy to reduce patient stress. The organization "Paws of Love" estimates that it has 180,000 volunteers who have qualified therapy dogs and who are generally willing to volunteer their time in emergency care. The benefits may include improved perception of wellness and less opioid use. Secondary effects may extend to improved interaction and lower use of chemical or physical restraings. As a further extrapolation, this secondary effect could lead to improved patient experience, and possibly lower risk of medical malpractice by improving the patient-provider relationship.(6, 7)

HYPOTHESES

The main study hypothesis is that patients with moderate to high anxiety will have reduced perception of anxiety when exposed to a therapy dog, compared with usual care.

SPECIFIC AIMS

1. Compare the effect of a single exposure to a therapy dog and handler on patient perception of anxiety using the FACES scale.

Exploratory aims

Test if a single exposure to a therapy dog and handler on patient perception of depression using the FACES scale.

Test if a single exposure to a therapy dog and handler reduces subsequent medication use for sedation and pain.

INCLUSION AND EXCLUSION CRITERIA

All study procedures will be performed in the emergency department at the Lois and Sydney Eskenazi hospital. Inclusion criteria require that the patient be at least 18 years of age, and that the emergency physician in charge of the patient agree that the patient has "moderate or greater anxiety". Exclusions include violent behavior, overt intoxication, any reported prior fear or adverse reaction to dogs.

ENROLLMENT/RANDOMIZATION

Participants will be recruited by verbal solicitation by qualified study personnel. Patients will be enrolled duing times when a hospital certified therapy dog and handler are present. Study

personnel will explain the study to the patient in the patient's room. After verbal consent, the patient will complete the baseline (T0) FACES scales of anxiety, depression and pain.{McKinley, 2008 #52;McKinley, 2004 #51} Patients who score a 0 on anxiety will be considered screen failures. To ensure matching, two patients will be studied on each shift: one assigned to see a therapy dog+ handler for 15-20 minutes and the other to usual care. These will follow a preprinted 1:1 block randomization schedule. Patients assigned to usual care will not see a therapy dog prior completing all study procedures.

STUDY PROCEDURES

We will ask physicians to record their perceptions of the patient's anxiety, depression and pain using the same scales.

We will then repeat the FACES scales 30 min after the departure of the dog or approximately 45 min after enrollment in usual care subjects (T1 measurement) and again as late as possible in the patient's stay (T2)

Patient demographic and medical data will be obtained in the emergency department at the time of enrollment and recorded on a REDcap form using an electronic tablet (see attachment). No PHI will be collected.

To understand more about the quality of the interaction between the dog, handler and the patient, we will ask handlers to keep field notes after sessions. They will be instructed to comment on verbal statements made by the patient, if the patient touched the dog, facial affect and other non-verbal communication made by the patient.

STATISTICAL CONSIDERATIONS

<u>Sample size</u>

The primary outcome is the change in reported anxiety on the FACES scale from T0 to T1, with the assumption that data will be normally distributed and the means will be the same at baselin. Therefore an unpaired t-test would be applicable. Extrapolating from prior work by Barker et al and Marcus, et al we set the clinically significant reduction in anxiety as requiring a greater than 2 point (20%) decrease in anxiety at T1 compared with usual care, expecting a standard deviation of 3.(8, 9) With $\alpha=0.05$ and $\beta=0.20$, this required 37 pairs. Accordingly, the sample was set at 40 per group with complete data.

Per protocol analysis: Aims 1 and 2 are tested by comparing mean or median values of the change in scales rom T0 to T1 and T2 (T1-T0 or ΔT) between the two interventions. Depending upon normality, we will either use an unpaired t-test or Mann Whitney U test to compare mean or medians from ΔT and to compare the mean or medians of T1. To examine for within group changes from T0 to T0, we will use either a paired t-test or a Wilcoxon rank sum test. If data are normally distributed or can be appropriately transformed to normal, we will also perform a 2-way repeated measures ANOVA to compare between groups for the change in perceived FACES scales. We will measure strength of correlation between patient and physician perceptions on the FACES scales with a Pearson's correlation coefficient or Spearman's rank coefficient as appropriate.

We will report the results of field notes in accordance with Field notes from dog handlers will be analyzed using commercial software (Nvivo Version 12.0.0.71, QSR International) to search for themes in patient verbal communication, changes in patient affect and changes in patient behavior from the start of the session to the end of the session.(19)

RECORD RETENTION AND PRIVACY

We will keep deidentified data indefinitely. Data will not be able to be reidentified to subjects.

References

1. Braun C, Stangler T, Narveson J, Pettingell S. Animal-assisted therapy as a pain relief intervention for children. Complementary therapies in clinical practice. 2009;15(2):105-9.
2. Marcus DA, Bernstein CD, Constantin JM, Kunkel FA, Breuer P, Hanlon RB. Impact of animal-assisted therapy for outpatients with fibromyalgia. Pain medicine (Malden, Mass). 2013;14(1):43-51.
3. Barker SB, Dawson KS. The effects of animal-assisted therapy on anxiety ratings of hospitalized psychiatric patients. Psychiatric services (Washington, DC). 1998;49(6):797-801.
4. Munoz Lasa S, Maximo Bocanegra N, Valero Alcaide R, Atin Arratibel MA, Varela Donoso E, Ferriero G. Animal assisted interventions in neurorehabilitation: a review of the most recent literature. Neurologia (Barcelona, Spain). 2015;30(1):1-7.
5. Havey J, Vlasses F, Vlasses P, Ludwig P, Hackbarth D. The Effect of Animal-Assisted Therapy on Pain Medication Use After Joint Replacement. Anthrozoos. 2014;27:361-9.
6. Smith DD, Kellar J, Walters EL, Reibling ET, Phan T, Green SM. Does emergency physician empathy reduce thoughts of litigation? A randomised trial. Emergency medicine journal : EMJ. 2016;33(8):548-52.
7. Kelm Z, Womer J, Walter JK, Feudtner C. Interventions to cultivate physician empathy: a systematic review. BMCMedEduc. 2014;14:219.
8. McConville J, McAleer R, Hahne A. Mindfulness Training for Health Profession Students-The Effect of Mindfulness Training on Psychological Well-Being, Learning and Clinical Performance of Health Professional Students: A Systematic Review of Randomized and Non-randomized Controlled Trials. Explore (New York, NY). 2017;13(1):26-45.
9. Mantzios M, Giannou K. When Did Coloring Books Become Mindful? Exploring the Effectiveness of a Novel Method of Mindfulness-Guided Instructions for Coloring Books to Increase Mindfulness and Decrease Anxiety. Frontiers in psychology. 2018;9:56.
