# Supplementary material for: Controlled clinical trial of canine therapy versus usual care to reduce patient anxiety in the emergency department
Source: PLoS One. 2019 Jan 9;14(1):e0209232. doi: 10.1371/journal.pone.0209232 (PMC6326463; doi:10.1371/journal.pone.0209232)
Supplement: S2 Table — (DOCX) [file pone.0209232.s007.docx]

| S2 Table. P values for depression scores | | | |
| --- | --- | --- | --- |
| Test | T0 | T1 | T2 |
| Unpaired t-test (+Dog vs. No Dog) | 0.282 | 0.04 | 0.069 |
| Mann Whitney U (+Dog vs. No Dog) | 0.221 | 0.04 | 0.064 |
|  | T0 vs. T1 | T0 vs. T2 |  |
| Paired t-test (+Dog) | <0.001 | 0.005 |  |
| Paired t-test (No Dog) | 0.21 | 0.01 |  |
|  |  |  |  |
|  |  |  |  |
|  |  |  |  |
