# Supplementary material for: Controlled clinical trial of canine therapy versus usual care to reduce patient anxiety in the emergency department
Source: PLoS One. 2019 Jan 9;14(1):e0209232. doi: 10.1371/journal.pone.0209232 (PMC6326463; doi:10.1371/journal.pone.0209232)
Supplement: S4 Table — (DOCX) [file pone.0209232.s009.docx]

| S4 Table. Performance of each therapy dog and handler | | | |
| --- | --- | --- | --- |
| Dog | Handler | Patients seen | median change T1-T0 |
| Aggie | Jamie | 1 | 0 |
| Bella | Emilie | 1 | 4 |
| Bonnie | Shelly | 2 | 0 |
| Cali | Christine | 2 | 3 |
| Charlie | Jessica | 1 | 4 |
| Dora | Valeri | 1 | 2 |
| Ed | Lilly | 1 | 0 |
| Gaia | Shelly | 13 | 3 |
| Izzy | Ed | 2 | 1 |
| Koda | Jessica | 1 | 4 |
| Luna | Sandy | 1 | 2 |
| Rex | Joe | 7 | 4 |
| Rocky | Doug | 1 | 0 |
| Sandy | Beth | 1 | 4 |
| Sarge | Jessica | 2 | 4 |
| Wilson | Jessica | 2 | 3 |
| Xena | Jamie | 1 | 8 |
